# The Institutional Worker

**Published:** 1913-02-15

**Authors:** 


THE
### INSTITUTIONAL WORKER
Being a Special Supplement to "The Hospital"

### BUREAU OF INFORMATION.

### Rules for Correspondents.

1. Every letter, on whatever subject, **must be accompanied by the coupon** to be cut from the back cover (inside page) of The Hospital, current issue, and must contain the name and address of the correspondent with pseudonym for publication if desired.

2. Replies by post cannot be given, save under exceptional circumstances at the Editor's discretion.

3. Letters from Approved Homes in reply to special needs published in the Bureau should state terms and full particulars, and be sent prepaid under cover to the Editor of the Bureau with name written across coupon for identification.

4. Proprietors of Homes which have not yet been entered on the List of Approved Homes, but have spare accommodation likely to suit special needs, are invited to write for an application form for registration. The fee for registration, which includes two announcements of the Home in the Bureau and other privileges, is 5s.

5. All communications to be addressed to the Editor of The Hospital, 28 Southamptan Street, Strand, London, W.C., and marked "Bureau of Information."

**INVITATION TO READERS.—The Editor is prepared to forward to applicants, without fee on either side, offers of accommodation from Homes on the Approved List suitable for medical and surgical cases, for the aged, for chronic or mental patients, for electrical treatment and massage, for convalescents and for children, at varying terms, in any required locality. Reduced terms can often be arranged. The special needs of any who may be sick or in difficulties are also made known in these columns free of charge.**

### Approved Homes Recently Added to List.

Note.—*No Home is added to the List without medical and other testimony to its good management.*

**Cornwall**.—Tregoddick, Flushing, Falmouth. Principal: Miss Kicke. For consumptive cases, who can be received in an advanced stage. Equable climate. Very sunny, cheerful situation, with view of harbour. Kindest care. Garden and shelter on edge of cliff.

**Harrogate.**—40 The Avenue, Starbeck. Principal: Miss Coulter. A very comfortable Home for medical, tubercular, chronic or slightly feeble-minded cases. Great experience and success with children. Healthy and bracing locality; garden.

### Needs and Helpers.

**Home for Chronic Invalid.**—Is there any Home near Liverpool or adjacent towns where a poor woman, crippled and almost helpless from rheumatism, could be received for a few shillings a week? She is entirely dependent on her two daughters, who are employed in a factory; very respectable. Prepaid letters to J. B. (150x).

**Home for Spinal Case.**—The patient is a poor respectable young woman, aged twenty-five, and is suffering from spinal and hip disease. She is able to do plain and fancy needlework and thus contribute a little towards her own support, but only 5s. a week can be paid for her. We know of no Home where she could be received for this sum, and fear the Poor-Law infirmary is the only resource. If sufficient money could be raised to make the amount up to 10s. 6d. a week we could place her, and we recommend your making an appeal and endeavouring to secure five subscribers who would give 1s. a week each for the relief of this very sad case.—E. P., Hollington (151x).

**Home Found.**—We are very glad that you have been able to receive our correspondent, who has suffered disfigurement, and we trust she will remain with you and become a useful worker. Many thanks for all the interest you have shown in our work, and for this second kind service rendered to the Bureau.—C. P. M. and E. G. B.

**Convalescent Home Wanted.**—The patient is a young married woman, suffering from gastritis, the second attack in a year; and a Home where care is given to diet is desired at a small cost. If you will write yourself for her to Mrs. Kilto, Free Convalescent Home, South Park, Reigate, you could probably procure her admittance there. It is very comfortably managed. If the sea is preferred, write to the Frederica Countess of Scarbrough's Convalescent Home, Skegness. The charge is 7s. 6d. a week.—A. L., Clapham Park.

### Training and Employment.

**Work for Injured Nurse-Attendant.**—Applicant has lost the middle finger of her right hand, and experiences some difficulty in getting employment in consequence. She has some knowledge of nursing and good references. Can any of our Homes offer her a berth at a small salary? She is a good needlewoman and experienced with babies.—Kathleen T.

**Training in Ceylon.**—The Colombo General Hospital is divided into two sections, each with its own nursing staff. Tell your friend to write to the Matron of the Paying Section, General Hospital, Colombo, and ask if there are facilities for training Europeans. There are ten sisters and nine Ceylonese nurses. The Pauper Section of the hospital is under the superintendence of the Reverend Mother Marie Angelina, who has only nuns under her. There is no other hospital in Ceylon with a training school for nurses. It would be much better to be trained in England. If you like to let us have further particulars about your friend's circumstances we shall be pleased to advise further.—Miss Z. M.

### Nursing in the Empire and Abroad.

**Colonial Nursing Association.**—Apply to the Secretary of the Colonial Nursing Association, Imperial Institute, South Kensington, S.W. Candidates must be fully trained and preferably gentlewomen; midwifery is an essential qualification. The age limit is 25 to 35. There is no special period of the year for applying. After the candidate's name is entered on the books for Colonial or Foreign work, she is expected to keep herself in readiness to go out when required.—E. S.

**The Need in Santiago.**—You might possibly procure information on this point from the Chilian Legation, 48 Grosvenor Square, London, S.W. Nurses would have to make their own clientèle and must be prepared to find it takes some time to get known and valued. There is no such prospect of instant employment as would make it safe to go out to Chile on the chance.—W. G. A. B.

**Male Nursing in Australia.**—Write to the Matron of the Public Hospital, Perth, Western Australia, or to the Matron of the Melbourne Hospital, Lonsdale Street, Melbourne. With the qualifications you name you would probably command higher fees working on your own account than in an institution. All fees are higher in Australia than in this country, but then the cost of living is higher also. Should you decide to emigrate you must go prepared to turn your hand to anything in the interval of working up a connection. You will not find a public accustomed to resort to massage as in England.—Medicus.

### Miscellaneous.

**Nursing Instructions in a Small Hospital.**—We are invited to afford information as to the correctness of your views in the following circumstances. Given a fever hospital of about forty beds with a permanent nursing staff of seven and excellent experienced charge nurses:—When the matron accompanies the medical superintendent or any visiting doctor to the wards, should not all directions relating to treatment of patients be given not to the matron, but to the charge nurse of the ward, the doctor writing them upon the patients' boards? In the case of the medical superintendent if the matron be at hand and does not visit the wards with him (although this is usually the case), he makes a point of seeing her afterwards and giving her details as to condition, discharge, and general treatment of the patients under his care.—In the case of a small hospital, such as that referred to, with a permanent and competent nursing staff, we fail to see any objection to the procedure described. Naturally the matron must have knowledge of the progress, diet, and treatment of the patients, but it is unnecessary for her to receive directly the doctor's instructions, which in any case will have to be carried out by the charge nurse of that ward, so that she cannot object. It saves repetition and possible error to give the nurse first-hand directions. Seeing that the matron probably interviews friends and answers letters, the medical superintendent's plan of seeing the matron afterwards (when she does not attend on his round) is a very good one, and only what is due to the matron. We assume the nurses employed are fully trained and certificated nurses of experience, or they would not be "charge nurses."—"Ichabod."

### Letter-Box.

**Communications have been received and attended to from:**—Miss A. S., St. Leonards-on-Sea; Nurse J. B., Reading; Nurse C., St. John's Wood.

**The Editor begs to acknowledge the receipt of communications with enclosures from:**—Miss Greenwood; Miss Coulter; "A Housekeeper"; A Northern Secretary; Miss Currie; Miss A. E. C.

**The Editor is much indebted for testimonials received in response to enquiries from:**—Dr. Carter Wilkinson; Mrs. Harrison; Mrs. Loft; Miss Peacock; G. Day, Esq.; B. W. Hilliard, Esq., J.P.; Dr. Boyle; Miss E. B. Smithers; Dr. Walter.

**Letters have been forwarded from:**—Mrs. D.; Miss G.; The Misses G.; Miss E. C.; Miss E.; Mrs. A.; M. E. M.

We have sent on your nice letter to the lady who visited you for us.—J. B., Maidstone.

The Editor is unable to introduce patients to any but the Homes on the Approved List.—Miss Castle.

Very sorry you have been disappointed of replies. We make a special request that this courtesy be extended to all letters received, but it is impossible to enforce it.—M. E. M.

We are making inquiries for you, and hope to give you some information on the subject shortly.—E. M. P.

By all means write freely to us, and we will do whatever we can.—Chronometer.

Your problem is very interesting, and we propose to set it for March. Delighted to have any suggestions in this direction.—A. E. C.

### Institution Facts and Figures.

Questions for February.

1. State the quantity of potatoes used in your institution during the month of December, and the price per cwt. paid for them. Enumerate all other kinds of vegetables, cooked or raw, which were served during this month. Was any of this produce grown in the institution gardens? Please state the number of beds.

2. Give the diet for convalescent patients in your institution, and state what it costs per week at the present prices.

The figures must be the result of actual observation and computation, not estimates. Either or both of the questions may be answered by each contributor. The best answers will be published, and for each contribution considered by the Editor to be of sufficient interest for publication payment of Five Shillings will be made.

The following rules must be observed by contributors to the Facts and Figures Column:—

1. Answers must be written on one side of the paper. They must bear the name and address of the sender and be accompanied by coupon to be cut from the back cover (inside page) of the current issue of The Hospital. A pseudonym must be chosen if the name is not to be published.

2. Answers must be addressed to the Editor of The Hospital, 28-29 Southampton Street, Strand, London, W.C.; they must be marked in the left-hand corner "Facts and Figures," and must be received before the end of the month.

### Housekeeping Queries.

**Lemon Puddings.**—Cream together 4 oz. of butter with an equal weight of sugar, add two eggs, well beaten; sift in 4 oz. of flour and the grated rind of one lemon; pour it into a buttered and floured basin and steam for two hours, and serve with lemon sauce.

Lemon Sauce.—1 oz. butter, 1 oz. sugar, ½ teacup of water and juice of one lemon; boil together for six minutes. A soupçon of cornflour stirred in makes a thicker syrup.

Another Lemon Pudding.—2 oz. flour, 2 oz. sugar, 1 lemon, 1 egg, 3 oz. breadcrumbs, 2 oz. suet, 1 small teaspoonful of baking-powder, and a little milk. Add the baking-powder to the flour, with the suet chopped finely, sugar, breadcrumbs, and the rind and juice of lemon. Then mix with the egg and milk, turn into a greased pudding mould, and steam for two hours.

For making boiled or steamed puddings you may substitute dripping for suet, and if this is beaten up first just as butter is creamed, and the sugar beaten with it, it makes the pudding beautifully light and spongy. In most institutions there is plenty of dripping, whereas suet has to be bought, so that it is also economical.—A. W.

**Soup at a Penny a Head.**—As you wish your soup to contain a little meat, the following recipe would suit your purpose. We are giving quantities for 45 persons as you desire. Put 4½ gallons of cold water into a boiler; add to it 6 lb. of mutton scraps or any butcher's pieces, chopping them into convenient sized pieces and removing and saving the fat for clarifying. When these ingredients have reached boiling-point, add 1½ oz. of salt and remove greasy scum. Cut one bunch each of carrots, turnips, and onions into discs after preparing them in the usual manner. Add these to the broth and 1½ lb. of washed pearl barley. Simmer gently for about six hours. Then take out as much bone as possible, ascertain if the seasoning is agreeable, and stir in a pennyworth of chopped parsley. The bones are more easily removed if the meat is put into a vegetable net and lowered into the water; it can then be quickly raised altogether. This amount should cost about 2s. 2d., or about ½d. per head, for the required numbers.—Parochial.

### Editor's Notices.

**Contributions :**

Contributions should be written, or preferably typed, on one side of the paper only, and all articles sent in are accepted upon the distinct understanding that they are forwarded to The Hospital only.

The Editor cannot undertake to return MSS. not used, but when a stamped directed envelope is enclosed the MSS. may be returned if a special request is made.

Accepted articles and paragraphs of news will be paid for after publication at the scale rate.

**Address :**

To prevent delay all contributions and letters on editorial business must be addressed exclusively to the Editor, "The Hospital," 29 Southampton Street, Strand, London, W.C. It is important that this regulation shall be strictly observed.

**Correspondence :**

Correspondence on all subjects is invited. The name and address of correspondents must be given as a guarantee of good faith, but not necessarily for publication.

**Special Articles :**

Special articles are invited, and questions, inquiries, and paragraphs upon all matters relating to the work, administration, and management of general, special, mental, fever, cottage and convalescent hospitals, sanatoria, homes, institutions, societies and organisations for the treatment or care of the sick, injured and dependants of all classes. Every matter affecting the interests and welfare of the staff of all grades working in these institutions will receive special consideration.

**Administrative Medicine, Research and Items of News :**

Special terms will be paid for approved articles on subjects in this wide field by experts who have made some section of it their special study and interest. We wish to give prominence to every movement tending to advance accuracy of diagnosis, efficiency in treatment, and the development of modern methods to eradicate disease and promote the welfare of the suffering.

**A Bureau of Information :**

We invite inquiries and applications for information and help in providing for that numerous class of sufferers who require special care or house-room which they cannot obtain in their own homes or secure for themselves. We cannot, however, prescribe, or recommend practitioners.

**Books for Review :**

Publishers are particularly requested to send advance proofs of any new books of importance whenever possible, as the Editor has made arrangements to publish immediate reviews on a new plan.

**Photographs, Plans, Blocks, and Illustrations :**

It is requested that wherever possible MSS. may be accompanied by illustrations in any of the above forms. The name of the sender and of the article to which it belongs should be written on the back of each photograph, drawing, or block for purposes of identification.

**Local Papers :**

Newspapers containing reports or news paragraphs should be marked and addressed to the Sub-Editor.

**The Coupon System :**

A coupon will be found at the bottom of the third inside page of the cover each week. This coupon must be attached to every question or inquiry to which an answer is desired in The Hospital.

### That Vacant Post.

As a medium for announcing vacancies in every department of Institutional Life, the exceptional value of *The Institutional Worker* is evidenced by the large number of such notices that appear week by week in this section of The Hospital. No more adequate proof of the usefulness of *The Institutional Worker* can be desired than the fact that the Responsible Officers of the most important Institutions in the United Kingdom employ its columns again and again whenever they have a vacancy to fill upon their staffs. It also provides exceptional facilities for every class of advertisement affecting Institutional Life and Work, and as a means of securing re-engagements in any section of the Institutional, Health, and Sanitary Services it is absolutely incomparable, since no other journal possesses such a wide and comprehensive circulation in these directions as The Hospital. If you are looking for a post an announcement of twenty-one words, costing the small sum of 1s., may save you many hours of anxious thought, and will certainly bring your requirement immediately before those most likely to need your services.

This form may be used for Appointments Wanted advertisements, and when filled up should be posted to

*The Manager*, The Hospital,
28 and 29 Southampton Street,
Strand, W.C.

Appointments Wanted, 21 words ... 1s. 0d.

| | | |
|---|---|---|
| | | |
| | | |
| | | |
| | | |
| | | |
| | | |
| | | |

### Manager's Notices.

**Letters relating to the publication, sale, and advertising departments of "The Hospital" must be addressed to The Manager, "The Hospital," The Hospital Building, 28 and 29 Southampton Street, London, W.C.**

**Subscriptions** may begin at any time, and are payable in advance. Cheques and Post-Office Orders should be crossed London County and Westminster Bank, Covent Garden Branch, and made payable to the Manager of The Hospital.

Rates of Subscription (including Postage).

| *United Kingdom.* | | *Foreign and Colonial.* | |
|---|---|---|---|
| Three Months ... ... | 2s. 0d. | Three Months ... ... | 2s. 6d. |
| Six Months ... ... | 4s. 0d | Six Months ... ... | 5s. 0d. |
| Twelve Months ... ... | 5s. 6d | Twelve Months ... ... | 8s. 8d. |

**Advertisements :**

To ensure insertion, all advertisements for The Hospital must reach the Manager not later than Wednesday morning in each week. For scale of charges see pages ix and 4.

**Remittances :**

**It is especially requested that remittances be made by Cheque or Postal Order, and in halfpenny stamps only when the amount is under sixpence.**

